# Adult Hirschsprung’s disease presenting as chronic constipation: a case report

**DOI:** 10.1186/s13256-023-03986-y

**Published:** 2023-07-05

**Authors:** Theresia Monica Rahardjo, Yeppy Arief Nurzaman, Janice Natalia, Indra Hapdijaya, Livia Devina, Hendrik Andrianto, Jeffrey Christian Mahardhika

**Affiliations:** 1 Faculty of Medicine, Maranatha Christian University, Suria Sumantri 65, Bandung, West Java 40164 Indonesia; 2 Unggul Karsa Medika Teaching Hospital, Taman Kopo Indah III Block H-1, Bandung, West Java 40218 Indonesia

**Keywords:** Hirschsprung’s disease, Constipation, Congenital, Adult, Case report

## Abstract

**Background:**

Hirschsprung’s disease is a congenital disorder identified by the absence of ganglion cells at the Meissner’s plexus of the submucosa and Auerbach’s plexus of the muscularis. This disease can be found in approximately 1 in 5000 live births. It is a congenital disorder that is rarely diagnosed in adults, where 95% of cases are diagnosed in infants aged under 1 year old. Here we present a rare case of adult Hirschsprung’s disease to enrich the body of knowledge  in diagnosing adult patients with chronic refractory constipation symptoms.

**Case report:**

An 18-year-old Indonesian woman came to the general surgery department of Unggul Karsa Medika Teaching Hospital with a defecating problem (constipation) since childhood. There was no history of her passage of meconium. A contrast enema study showed that the sigmoid colon was dilated and the rectum was narrowed, with rectosigmoid index < 1. With these findings, it was suspected that the patient may have ultra-short segment Hirschsprung’s disease. The patient was then referred to the digestive surgery department of referral hospital for surgical treatment.

**Conclusion:**

In adult patients presenting with history of constipation since childhood, it is necessary to consider the possibility of Hirschsprung’s disease that was not diagnosed in early childhood. Hirschsprung’s disease in adults is usually a short or ultra-short aganglionic segment because it shows relatively mild symptoms. Surgical removal of the aganglionic segment of the gut is the definitive treatment for Hirschsprung’s disease.

## Background

Hirschsprung’s disease is a congenital disorder identified by the absence of ganglion cells at the Meissner’s plexus of the submucosa and Auerbach’s plexus of the muscularis. It is usually characterized by nonspecific symptoms such as chronic constipation [1]. Hirschsprung’s disease can be found in approximately 1 in 5000 live births [2]. Since it is a rare disease, literature study is often conducted without gender-specific analysis and only including a small number of patients [3]. The diagnosis is made before the age of 1 month in 65% of the total cases and before the age of 1 year in 95% of the total cases [1]. Rectal touch findings might show a tight anal sphincter and explosive discharge of gas and stool. Despite the fact that most patients are identified in their infancy and the early stages of their lives, there are some rare cases that may not be detected until the age of adolescence or adult [4]. This report presents a rare case of chronic constipation of an adult who was later diagnosed as Hirschsprung’s disease.

## Case presentation

An 18-year-old Indonesian woman came to the general surgery department of Unggul Karsa Medika Teaching Hospital with a main complaint of a defecating problem (constipation) since childhood. She said that her defecation frequency is once or twice a month. She has not taken any medical interventions in any hospital or clinic except over-the-counter drugs such as cathartic drugs. She was unaware of her past history of passage of meconium. There was no history of growth or development problems. Physical examinations conducted on the patient showed her abdomen was slightly bloated, and rectal touch showed a collapsed ampullae.

**Fig. 1:**
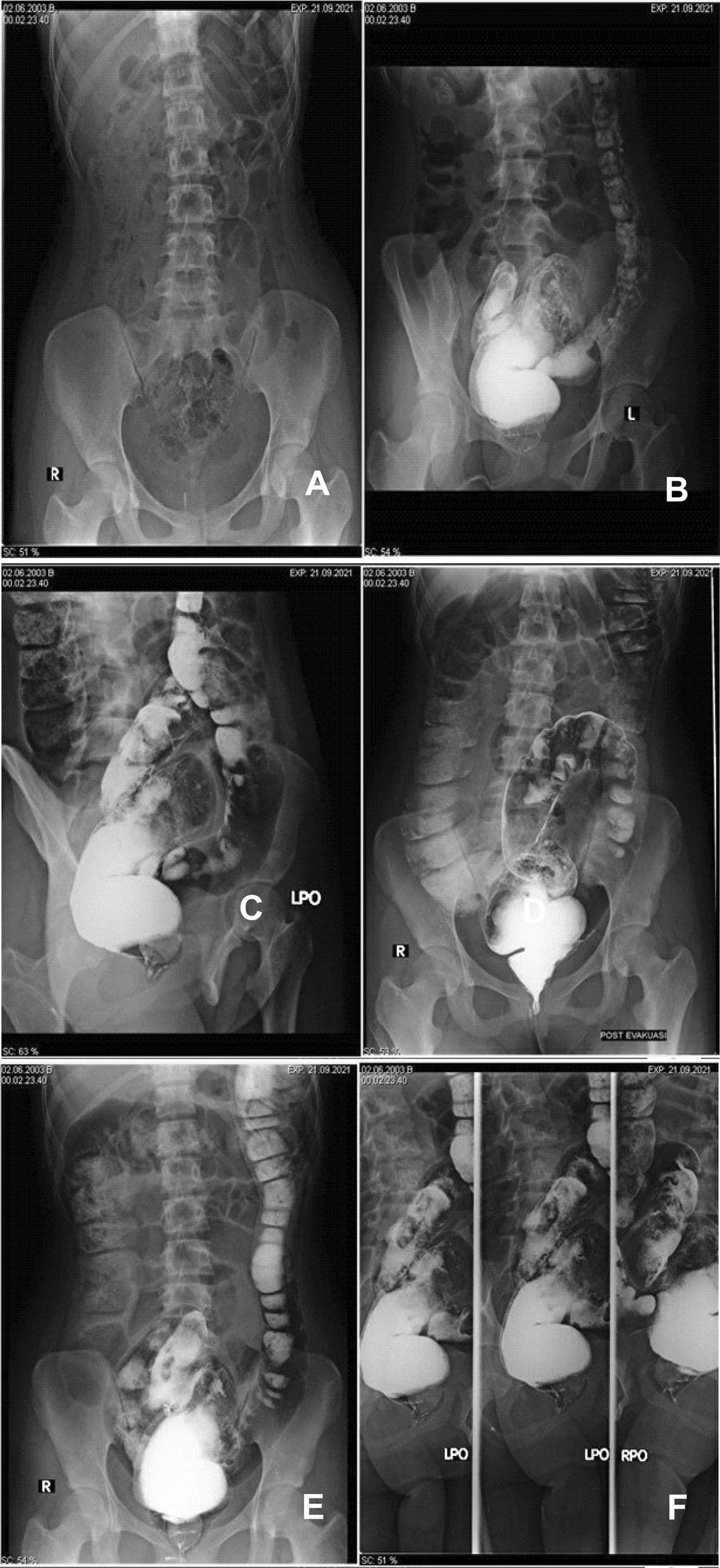
**A** Abdominal plain radiograph. This figure revealed that the air distribution in the colon and small intestine is increased with coiled spring appearance. There was neither stepladder appearance nor subdiaphragmatic free air. **B**–**F** Contrast enema study radiograph. The study showed that the sigmoid colon was dilated and the rectum was narrowed, with rectosigmoid index less than 1 and irregular rectosigmoid mucosa

Plain abdominal radiograph revealed that the air distribution in the colon and small intestine was increased with coiled spring appearance (Fig. 1A). There was neither stepladder appearance nor subdiaphragmatic free air. A contrast enema study was performed and showed that the sigmoid colon was dilated and the rectum was narrowed with rectosigmoid index less than 1 and irregular rectosigmoid mucosa. The study also showed neither rat tail appearance nor filling defects (Fig. 1B–F). With these findings, it was suspected that the patient may have ultra-short segment hypo-ganglionic type of Hirschsprung’s disease. As we do not have a digestive surgery consultant to perform the surgery, we performed rectal washout using laxative agents. The patient felt better after she could defecate, and we then referred her to the nearest referral hospital for surgical intervention. However, owing to economic problems, the patient could not afford surgery and thus only used laxative agents once a week. The patient said laxative can relieve constipation and help to improve her quality of life, even though surgery is the main treatment for her condition.

## Discussion

Constipation is a common disorder that usually refers to persistent, infrequent defecation, difficult stool passage with pain and stiffness [5]. Chronic constipation in adults is the sixth most common gastrointestinal symptom, with global prevalence of 15%. Chronic constipation affects women more than men, with a median female-to-male ratio of 1.5:1. The prevalence is also higher in non-Caucasians group compared with Caucasians. It can be either primary or secondary [6].

The pathophysiology and differential diagnosis of chronic constipation are multifactorial and include iatrogenic, organic stenosis, endocrine or metabolic disorders, neurological disorders, enteric neuropathies, myogenic disorders, colon disease (such as irritable bowel syndrome or diverticulitis), and anorectal disorders [5, 7]. Careful history-taking and physical examination should be performed, including duration of symptoms, frequency and consistency of stool, size of the stool, sensation of incomplete stool evacuation, straining, and digital rectal examination [5, 8]. After the initial history and physical examination, a series of tests should be performed to exclude underlying diseases. Barium enema, endoscopy, anorectal manometry, and balloon expulsion test can be considered to diagnose chronic constipation [5].

Hirschsprung's disease is characterized as the total nonexistence of ganglion cells within the colonic wall and an absent recto-anal inhibitory reflex. This is the most frequent finding within the gastrointestinal neuromuscular disorder, a group of disorders that also includes hypoganglionosis, ganglioneuromatosis, intestinal neuronal dysplasia, myopathies, and abnormalities of the interstitial cells of Cajal. It is known in 2% of patients, a chief complaint of chronic constipation is caused by the disease. This symptom of chronic constipation will show up as a result of short aganglionic segment in the descending part of the colon less than 10 cm in length [2].

Hirschsprung's disease is one of the main causes of intestinal obstruction in infancy. Diagnosis and treatment are conducted at an early age, in the first 5 years of life in more than 90% of cases. Consequently, the diagnosis of Hirschsprung’s disease in adulthood is rare [9]. The classic clinical symptoms including abdominal distension (in more than 90% cases), vomiting (more than 85% cases), which may be bilious, and failure to pass meconium during the first 24 h of life (more than 60% cases) [10].

Adult Hirschsprung’s disease is commonly misdiagnosed as refractory constipation. The absence of intramural ganglion cells in the affected segment of the colon is the underlying mechanism of refractory constipation in Hirschsprung’s disease in both infants and adults. Mild Hirschsprung’s disease in early life might go undetected due to the proximal colon compensating for the nonmobile colon segment, then presenting as chronic refractory constipation in adulthood. When this happens, most patients usually use cathartic agents to alleviate this symptom. However, at some point, this condition can deteriorate into distal colon obstruction, then the patient will experience rapidly worsening constipation or even acute intestinal obstruction [11].

Once Hirschsprung’s disease is clinically suspected, imaging studies, anal manometry, and full-thickness rectal biopsies are usually performed to confirm the diagnosis [2]. Plain abdominal radiograph is usually the first investigation. It might demonstrate grossly distended large bowel, with possible absence of stool in the distal colon or rectum. However, the water-soluble contrast enema, which has been used for more than 50 years to diagnose Hirschsprung’s disease, is a radiologic examination of choice. It shows a transition zone between a narrow distal aganglionic bowel segment and distended proximal ganglionated bowel, which is considered to be the most accurate radiologic feature of Hirschsprung’s disease [12].

A case similar to that reported herein was reported in Bali, Indonesia: a 13-year-old girl who had chronic refractory constipation and abdominal distension since birth. The patient only defecated once a month. Histopathological examination was performed, and the result confirmed that the distal rectum part was aganglionic. Therefore, the patient was diagnosed with ultra-short segment Hirschsprung's disease [13]. Another case of adult Hirschprung’s disease was reported by Soussan *et al*. The patient was a 20-year-old male with history of chronic constipation who presented with inability to evacuate stool and gas along with abdominal pain. Abdominal CT scan showed dilated colon and intestine. Surgical treatment was done, and histological findings showed aganglionic part of sigmoid, thus confirming the diagnosis of Hirschprung’s disease [14].

Based on the length of the aganglionic segment of the colon, Hirschsprung's disease is classified into four categories. The first one is short aganglionic segment (75–80% of cases) in which the aganglionic segment is present in the distal sigmoid colon and rectum. The second one is long aganglionic segment (10% of cases) in which the aganglionic colon segment is present from distal sigmoid colon and rectum until splenic flexure. The third one is the rarest form of Hirschsprung's disease and has the most severe clinical course, which is total colonic aganglionosis or involving the entire colon (5% of cases). The last one is called the ultra-short aganlionic segment, which involves only the distal rectum and the anal canal above the pectinate line. Hirschsprung’s disease in adults usually presents as short aganglionic segment or ultra-short aganglionic segment, thus showing relatively mild symptoms, especially in the early stages of life [13].

Intravenous fluid resuscitation, decompression method with nasogastric tube insertion and rectal washouts, along with antibiotic treatment when indicated, for example when perforation of abdominal organs is suspected, are the initial treatments for adult Hirschsprung’s disesase. Meanwhile, surgical removal of the aganglionic segment remains the definitive treatment for this disease [15].

The surgical approach for Hirschsprung’s disease is determined by the length of the achalasic zone, the length and reversibility of colonic dilation, the nutritional status of the patient, and the experience of the operator. Trans-anal myectomy is primarily used to remove the spasm from the aganglionic zone when it is very short. Sigmoid rectal resection with colo-anal anastomosis and Swenson’s procedure is used to remove the aganglional part of the rectum and the irreversibly distended part of the upstream colon [11].

The Duhamel technique is based on the principle of a short circuit of the aganglionic zone, lowering the healthy colon behind the diseased rectum left in place. This technique is performed when rectal dissection is very difficult to achieve and has morbid consequences to the patient. Another technique is the Soave’s procedure, which is resecting the distended colon and the upper part of the pathological rectum, but then the resection stops before approaching the lower rectum. The ganglionic colon is then pulled through the rectum, from which the mucosa has been removed. This surgical technique has a degree of difficulty that is related to the length of the remaining ganglionic colon of the patient [16].

We report herein a rare case of adult Hirschsprung’s disease with history of chronic refractory constipation since childhood. While our case report did not present how the definitive treatment was done or its result owing to the lack of digestive surgeon in our hospital, this case report suggests the necessity to workup towards Hirschsprung’s disease when encountering patients with chronic refractory constipation. Hirschsprung’s disease in adults is usually caused by short aganglionic segment of distal colon or ultra-short aganglionic segment because those types will show relatively mild symptoms, thus leading to late diagnosis of the disease [13]. Careful history-taking, physical examination, and the usage of simple radiology modalities such as abdominal plain radiograph and contrast enema study can be very useful in diagnosing adult Hirschsprung’s disease. Radiological studies showed signs of distal colon obstruction as there was increased air distribution in the colon and coiled spring appearance, then the contrast enema study showed dilation of sigmoid colon and narrow rectum with rectosigmoid index less than 1 and irregular rectosigmoid mucosa. These findings thus directed our clinical judgement to the diagnosis of Hirschsprung’s disease.

Initial treatment to decompress the abdomen was necessary to alleviate the symptoms, using nasogastric tube and rectal washout using laxative agents. However, the definitive treatment of surgery using one of the techniques stated above has to be done.

## Conclusion

In adult patients presenting with history of constipation since childhood, it is necessary to consider the possibility of Hirschsprung’s disease which was failed to be diagnosed in early childhood. Hirschsprung’s disease in adults is usually a short or ultra-short aganglionic segment because it shows relatively mild symptoms. Surgical removal of the aganglionic segment of the colon is definitive treatment for Hirschsprung’s disease.

## Data Availability

The data and materials from this study are available from the corresponding author.

## References

[1] Lotfollahzadeh S, Taherian M, Anand S. Hirschsprung Disease. In: StatPearls. Treasure Island (FL): StatPearls Publishing; 2022 Jan. https://www.ncbi.nlm.nih.gov/books/NBK562142/. Accessed 3 Dec 2022. 32965813

[2] Reategui CO, Spears CA, Allred GA (2021). Adults Hirschsprung's disease, a call for awareness. A case report and review of the literature. Int J Surg Case Rep..

[3] Granéli C, Dahlin E, Börjesson A, Arnbjörnsson E, Stenström P (2017). Diagnosis, symptoms, and outcomes of Hirschsprung's disease from the perspective of gender. Surg Res Pract.

[4] Howsawi A, Bamefleh H, Al Jadaan S (2019). Clinicopathological characteristics of Hirschsprung’s disease with emphasis on diagnosis and management: a single-center study in the Kingdom of Saudi Arabia. Glob Pediatr Health..

[5] Forootan M, Bagheri N, Darvishi M (2018). Chronic constipation: a review of literature. Medicine (Baltimore).

[6] Bharucha AE, Lacy BE (2020). Mechanisms, evaluation, and management of chronic constipation. Gastroenterology.

[7] Basilisco G, Coletta M (2013). Chronic constipation: a critical review. Dig Liver Dis.

[8] Wald A (2016). Constipation: advances in diagnosis and treatment. JAMA.

[9] Adamou H, Amadou Magagi I, Habou O, Adakal O, Aboulaye MB, Robnodji A, James Didier L, Sani R, Abarchi H (2019). Diagnosis and surgical approach of adult Hirschsprung's disease: about two observations and review of the literature. Case Series. Ann Med Surg (Lond).

[10] Kyrklund K, Sloots CEJ, de Blaauw I, Bjørnland K, Rolle U, Cavalieri D, Francalanci P, Fusaro F, Lemli A, Schwarzer N, Fascetti-Leon F, Thapar N, Johansen LS, Berrebi D, Hugot JP, Crétolle C, Brooks AS, Hofstra RM, Wester T, Pakarinen MP (2020). ERNICA guidelines for the management of rectosigmoid Hirschsprung's disease. Orphanet J Rare Dis.

[11] Haider T, Guddati H, Nawaz W, Hertan H (2018). Adult hirschsprung disease (HD): an uncommon cause of chronic refractory constipation in adults. Am J Gastroenterol.

[12] Vlok SSC, Moore SW, Schubert PT, Pitcher RD (2020). Accuracy of colonic mucosal patterns at contrast enema for diagnosis of Hirschsprung disease. Pediatr Radiol.

[13] Agustina K, Margiani NN, Anandasari PPY, Mahastuti NM (2021). Constipation that needs attention: late Hirschsprung disease. Intisari Sains Medis.

[14] Soussan H, Jabi R, Ouryemchi M, Haddadi Z, Bouziane M (2021). Hirschsprung's disease in adults revealed by an occlusive syndrome. Cureus..

[15] Shair KA, Edwards E (2020). Hirschsprung's disease in an adult. Am J Med.

[16] Lupon E, Labbe F, Nini E, Sondji S (2019). Hirschsprung disease in an adult with intestinal malrotation and volvulus: an exceptional association. J Med Case Rep.

